# Mouse Models for Methylmalonic Aciduria

**DOI:** 10.1371/journal.pone.0040609

**Published:** 2012-07-09

**Authors:** Heidi L. Peters, James J. Pitt, Leonie R. Wood, Natasha J. Hamilton, Joseph P. Sarsero, Nicole E. Buck

**Affiliations:** 1 Metabolic Research, Murdoch Childrens Research Institute, Department of Paediatrics University of Melbourne, Royal Children's Hospital, Parkville, Australia; 2 VCGS Pathology, Murdoch Childrens Research Institute, Royal Children's Hospital, Parkville, Australia; 3 Cell and Gene Therapy, Murdoch Childrens Research Institute, Department of Paediatrics, University of Melbourne, Royal Children's Hospital, Parkville, Australia; University Hospital Vall d'Hebron, Spain

## Abstract

Methylmalonic aciduria (MMA) is a disorder of organic acid metabolism resulting from a functional defect of methylmalonyl-CoA mutase (MCM). MMA is associated with significant morbidity and mortality, thus therapies are necessary to help improve quality of life and prevent renal and neurological complications. Transgenic mice carrying an intact human MCM locus have been produced. Four separate transgenic lines were established and characterised as carrying two, four, five or six copies of the transgene in a single integration site. Transgenic mice from the 2-copy line were crossed with heterozygous knockout MCM mice to generate mice hemizygous for the human transgene on a homozygous knockout background. Partial rescue of the uniform neonatal lethality seen in homozygous knockout mice was observed. These rescued mice were significantly smaller than control littermates (mice with mouse MCM gene). Biochemically, these partial rescue mice exhibited elevated methylmalonic acid levels in urine, plasma, kidney, liver and brain tissue. Acylcarnitine analysis of blood spots revealed elevated propionylcarnitine levels. Analysis of mRNA expression confirms the human transgene is expressed at higher levels than observed for the wild type, with highest expression in the kidney followed closely by brain and liver. Partial rescue mouse fibroblast cultures had only 20% of the wild type MCM enzyme activity. It is anticipated that this humanised partial rescue mouse model of MMA will enable evaluation of long-term pathophysiological effects of elevated methylmalonic acid levels and be a valuable model for the investigation of therapeutic strategies, such as cell transplantation.

## Introduction

Methylmalonyl-CoA mutase (MCM, EC 5.4.99.2) catalyses the conversion of methylmalonyl-CoA to succinyl-CoA. Deficiency of the enzyme leads to accumulation of methylmalonyl-CoA and to a lesser extent propionyl-CoA, and is termed methylmalonic aciduria (MMA, OMIM 251000). This rare inherited disorder of organic acid metabolism is inherited in an autosomal recessive manner and occurs with an incidence of approximately 1∶120,000 [1]. MMA patients that have defects in the MCM gene (*MUT*) have traditionally been categorised into mut^0^ and mut^-^ forms based on complete or partial absence of functional apoenzyme respectively [2].

Those with the mut^0^ form usually present within the first days to month of life with progressive severe metabolic acidosis accompanied by poor feeding, vomiting, lethargy, hypotonia and secondary metabolic disturbances causing hyperammonemia, hyperglycinaemia, hypoglycaemia and ketosis [3], [4]. Those with the mut^-^ forms may have a less acute presentation, with poor growth and failure to thrive during the first one to two years of life.

Poor growth and nutrition represent a major problem associated with the disorder. Individuals often have significant anorexia and may require periods of prolonged nasogastric feeding and are often below the mean for height [5], [6], [7], [8], [9], [10]. Other complications include neutropenia and anaemia through to secondary marrow suppression [11], [12], [13], [14]. Pancreatitis and cardiomyopathy have been variably reported [15].

The mouse mutase locus (*Mut*) was mapped using a human cDNA clone to chromosome 17 [16]. The mouse gene structure was determined in 1990 by Wilkemeyer *et al.* [17] and was shown to have 94% amino acid sequence identity to the human and 57% identity with *MUTB* of *P. shermanii*. *Mut* codes for a protein of 748 amino acids with a 30 amino acid mitochondrial targeting leader sequence. The greatest region of divergence is within the mitochondrial targeting sequence where there is only 69% identity to human.

The mouse MCM was shown to have similar activity to the human [17]. Tissue specific studies of mRNA levels identified greatest levels in kidney, followed by heart, brain, liver, muscle and least in lung and spleen [18]. There was a log linear relationship between total enzyme activity and mRNA, but no relationship between holoenzyme activity and mRNA levels.

Previously we developed a knockout mouse model for MMA by disrupting the mouse mutase gene (*Mut*) within the critical CoA binding domain using gene-targeting techniques [19]. Heterozygous knock-out animals (*Mut^+/−^*) were phenotypically normal and had normal growth and fertility. Homozygous progeny (*Mut^−/−^*) (produced by heterozygous intercrossing) were born normally, however became sick after 15 h and did not survive beyond 24 h of age. The reduced amount of mutase activity resulted in increased methylmalonic acid in urine, and elevated blood propionylcarnitine (C3) levels, similar to the human condition [19].

Development of cellular genomic reporter assays for MMA have allowed the identification of compounds that have the potential of modulating the expression of MCM which provides new approachs for the treatment of methylmalonic aciduria [20], [21].

Whilst our knockout mouse model provides a powerful tool by which to reproduce a human disease phenotype and has the potential to provide important insights into mechanisms of disease pathology, it does not provide a model which can be easily manipulated for trialling new compounds or other methods of treatment. Gene knockout is clearly a quite different mechanism to the presence of a gene carrying a point mutation and precludes the investigation of treatments aimed at increasing residual activity. Furthermore, there may be treatments that are specific to the human locus. One approach to circumvent this has been the development of humanised transgenic mice.

Here we describe the development of several mouse lines carrying the human MCM gene and the partial ‘rescuing’ of the neonatal lethality observed in homozygous knockout mice to produce a humanised transgenic MMA mouse model.

## Results

### Transgenic Mouse Models

**Figure 1:**
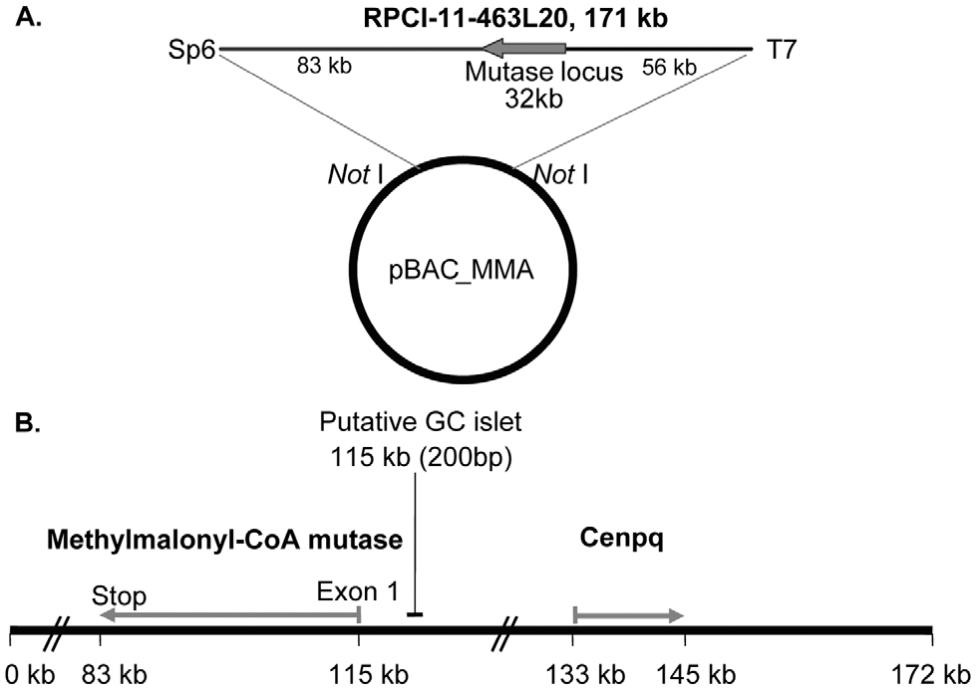
Diagram of pBAC_MMA showing the size of the insert and the relation of the methylmalonyl-CoA mutase locus to flanking upstream and downstream sequence (A). (**B**) Two intact genes are contained within pBAC_MMA. The methylmalonyl-CoA mutase and centromere protein Q (Cenpq) loci are divergently transcribed and share a putative 200 bp CpG island.

Six separate founder mouse lines containing the MMA_BAC (**Figure 1**) were developed on a C57BL/6 background. Of these mouse lines, four were found to transmit the intact transgene (**Table 1**). The founder line with one copy of the transgene did not have the complete transgene, whilst a mouse with three copies of the transgene was unable to transmit the transgene to the next generation.

**Table 1:** Summary of results from six transgenic founder lines.

MouseLine	Integration	Integrity of transgene	Founder copy number	Progeny copy number	Transmitting	Generations bred
**A**	single	intact	**0.6**	**2**	Yes	13
**B**	single	5′ and 3′ ends missing	**1**	N/A	N/A	0
**C**	single	intact	**2**	**4**	Yes	16
**D**	single	intact	**2**	**5**	Yes	3
**E**	single	intact	**3**	N/A	No	0
**F**	single	intact	**6**	**6**	Yes	4

**Figure 2:**
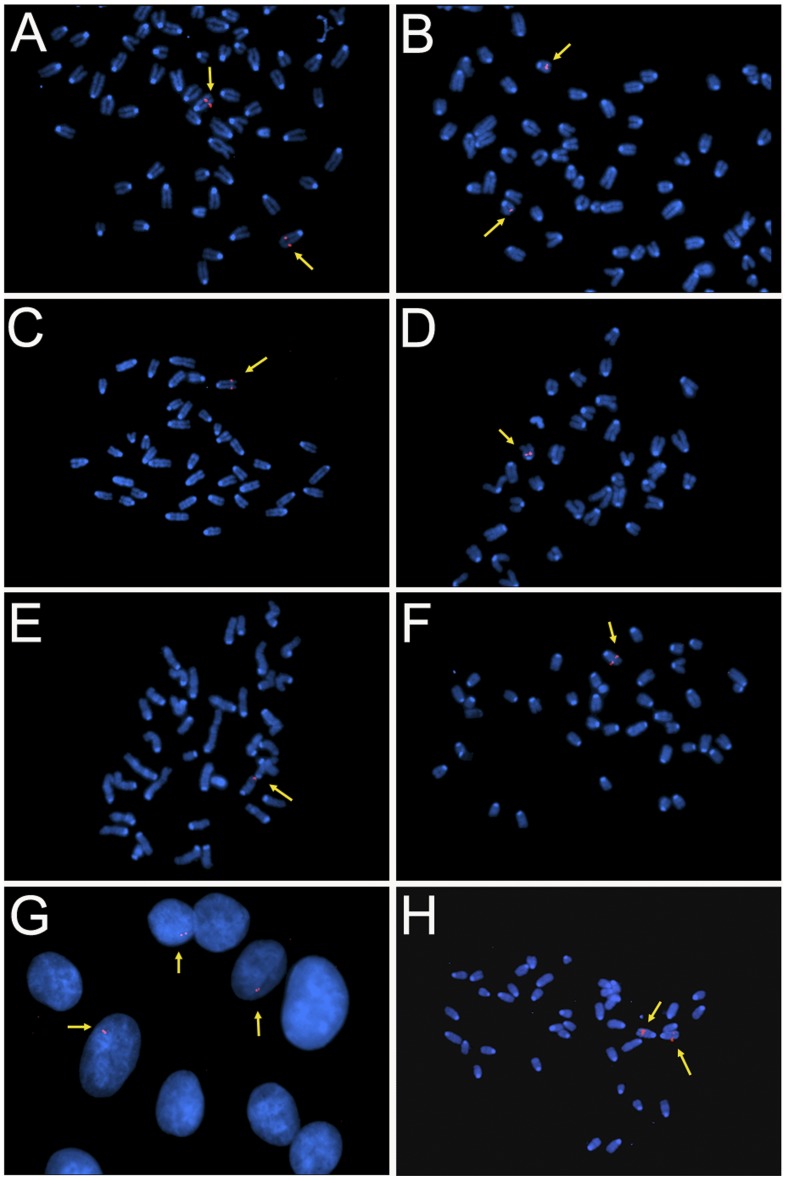
Fluorescent *in situ* hybridisation study on fibroblasts probed with digoxigenin labelled BAC RPCI-11-463L20. Yellow arrows indicate where the transgene (pink spots) has been detected. **A–F**: 0.6-, 1-, 2-(Mouse C), 2-(Mouse E), 3- and 6-copy mouse founder lines respectively. The presence of only one signal per diploid number establishes the presence of a single integration site of the mutase transgene. **G:** The 0.6-copy line (Mouse A) did not have a signal in all cells of the interphase spread, suggesting the possibility of mosaicism. **H:** Metaphase spread from a 2-copy homozygous mouse (0.6-copy founder line) showing two sites where the transgene was detected.

Fluorescent *in situ* hybridisation analysis showed the presence of a single signal in each diploid metaphase thereby providing confirmation of a single integration site in each transgenic line (**Figure 2**). The founder lines contained 0.6 to 6 copies of the transgene, whilst the progeny of the transmitting lines had two to six copies of the transgene (**Table 1**). Mosaicism in the founders would explain the difference obtained between founders and progeny.

The mouse lines in which the progeny carried 2 copies (*MUT^2h^*) (Mouse line A) or 4 copies (*MUT^4h^*) (Mouse line C) of the transgene were bred to more than ten generations. They had a mean litter size of 6.6 and 3.5 pups respectively, with a female to male ratio of about 1∶1. The 4-copy line (*MUT^4h^*) had a significantly smaller than normal litter size suggesting the transgene had interrupted a gene influencing reproduction. The transmittance for the lines was approximately 50% as expected from hemizygous by C57BL/6 breeding pairs.

The mouse lines in which the progeny carried 5 (*MUT^5h^*) or 6 (*MUT^6h^*) copies of the transgene had a mean litter size of 5.7 and 6.1 pups respectively, with a female to male ratio of about 1∶1. The transmittance for the lines was 50% as expected from hemizygous by C57BL/6 breeding pairs.

**Figure 3:**
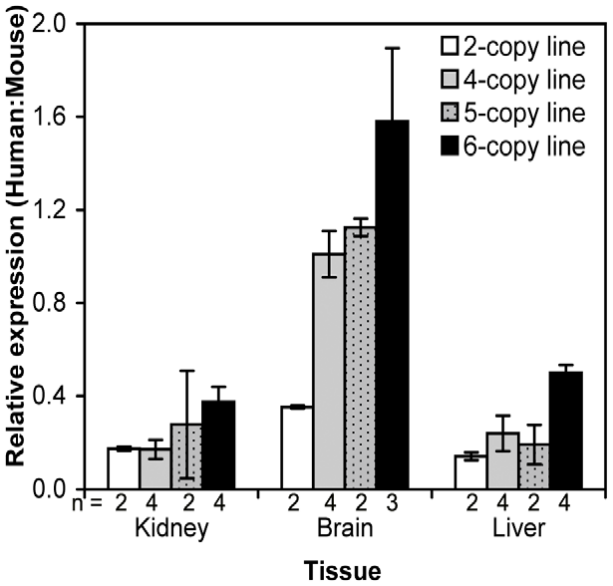
Relative gene expression analysis of the human *MUT* transgene compared to mouse *Mut* gene in different tissues from the four independent transgenic progeny mouse lines with various copy numbers of the human *MUT* gene. Data presented as mean ± SEM.

Human *MUT* mRNA was expressed in kidney, brain and liver (**Figure 3**). Whilst major differences in the human:mouse ratio were observed between tissues, the overall pattern of expression was similar in all four lines. The human:mouse ratio was least in the kidney, where human mutase expression was only 17–37% the level of mouse mutase expression. Slightly higher levels occurred in the liver and highest levels were observed in the brain, where the expression reached up to one and a half times the mouse, depending on transgene copy number (**Figure 3**).

### Complementation of Mutase Knockout and 2- and 4-copy Rescue Mice

The 2-copy (*MUT^2h^*) transgenic mice (Mouse line A) were mated against heterozygous knockout mice (*Mut^+^* ^/−^) to produce hemizygous (*Mut^−/−^MUT^2h^*) and homozygous (*Mut^−/−^MUT^2h/2h^*) ‘partial rescue’ mice (mice which had no endogenous mouse mutase gene (*Mut*) and one or two copies of the 2-copy human mutase gene (*MUT^2h^*)). The mean litter size was 7 pups, with a female to male ratio of 1∶1 and expected transgene transmittance according to Mendelian inheritance.

The hemizygous 2-copy partial rescue mice (*Mut* ^−/−^ *MUT* ^2h^) were born normally and were indistinguishable from their litter mates (*Mut^+/−^*, *Mut^+/+^, Mut^+/−^MUT^2h^* or *Mut^+/+^MUT^2h^*) at birth. They were of comparable size and weight, were active, vigorous and fed well. They survived the 24 hour neonatal period with no symptoms, unlike *Mut^−/−^* mice [19].

**Figure 4:**
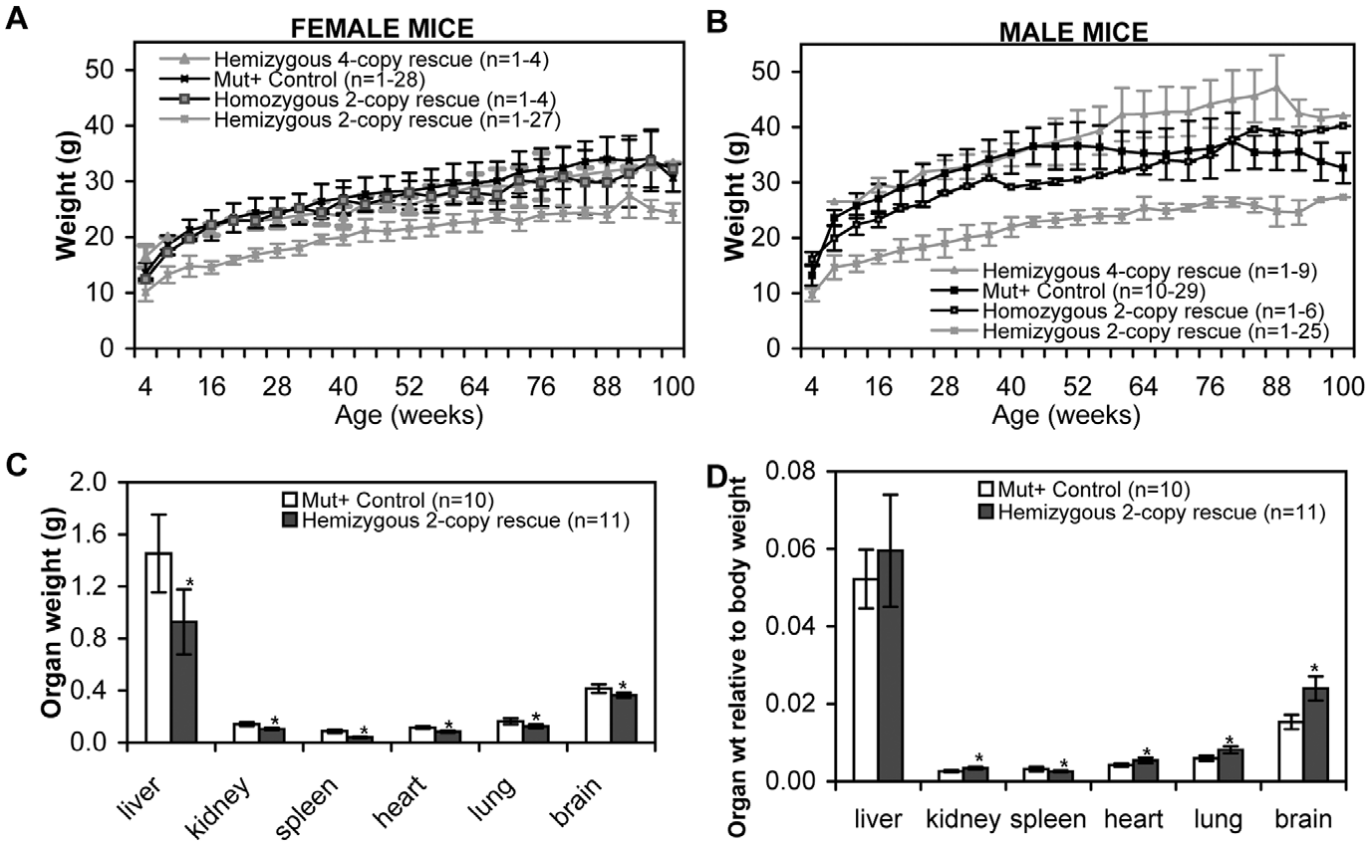
Comparison of weights of the 2-copy and 4-copy mouse lines. Body weight over two years (A) female mice and (B) male mice. Organ weight of four month old mice (C) actual weight and (D) organ weight relative to body weight, where the mean body weight for four month old *Mut^+^* controls (*Mut^+/−^* or *Mut^+/+^*) was 30.0±6.4 g and for hemizygous 2-copy partial rescue was 15.4±1.6 g. Data presented as mean ± SEM. *p<0.05 hemizygous 2-copy partial rescue vs *Mut* ^+^ mouse tissue.

**Figure 5:**
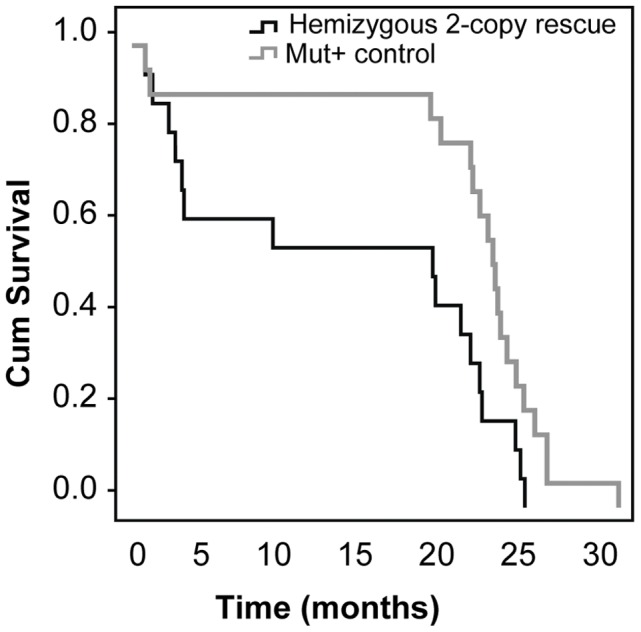
Kaplan Myer curve comparing survival rates for rescue mice relative to control mice over a 30 month period. Rescue mice have a higher earlier loss in the first 6 months, which then plateaus and follows the loss seen in control mice.

The hemizygous 2-copy partial rescue pups remained indistinguishable in size from litter mates (*Mut^+/−^MUT^2h^* or *Mut^+/+^MUT^2h^*) until around the third week of life, when an increasing difference in weight became noticeable (**Figure 4**). There was a significant difference in weight for both male and female hemizygous 2-copy rescue mice compared to unaffected mice. Their behaviour however was generally very similar to litter mates; they remained active, inquisitive and had similar movements and speed. Within the first six months of life these rescue mice were prone to having episodes when they appeared sick (decreased movement, hunched over and ruffled fur). These episodes generally resolved by providing the mice with additional mushy food and seed. After the first 6 months these episodes were infrequent and a normal life span could be achieved (**Figure 5**).

Homozygous 2-copy partial rescue mice (*Mut* ^−/−^ *MUT* ^2h/2h^) were born normally and were indistinguishable from litter mates. They were significantly larger than hemizygous partial rescue mice (*Mut^+/+^MUT^2h^*), but were slightly smaller than *Mut^+/−^* or *Mut^+/+^* litter mates, suggesting an intermediate phenotype (**Figure 4**).

Liver, kidney, spleen, heart, lung and brain weights of hemizygous 2-copy partial rescue mice (*Mut* ^−/−^ *MUT* ^2h^) at four months of age were compared to litter mate *Mut^+/−^* or *Mut^+/+^* control mice (**Figure 4**). Whilst the actual weights of the organs was significantly lower (*p*<0.05) for hemizygous 2-copy partial rescue mice compared to litter mate *Mut^+/−^* or *Mut^+/+^* mice, when the weights were compared to total body weight (hemizygous 2-copy partial rescue mice 15.4±1.6 g, *Mut^+/−^* or *Mut^+/+^* mice 30.0±6.4 g) there is a significant increase (*p*<0.05) in the relative weight of the kidney, heart, lung and brain of hemizygous 2-copy partial rescue mice. Spleen relative weight was significantly less in hemizygous 2-copy partial rescue mice, whilst the liver was not significantly different in relative weight to control mice.

The 4-copy (*MUT* ^4h^) transgenic mice (Mouse line C) were also mated against heterozygous knockout mice to produce hemizygous (*Mut* ^−/−^ *MUT* ^4h^) and homozygous (*Mut* ^−/−^ *MUT* ^4h/4h^) ‘rescue’ mice. They had a mean litter size of 3 pups with a loss rate of 33% prior to wean. The female to male ratio was 1∶1, whilst the transgene transmittance was at the expected ratio. The hemizygous 4-copy partial rescue mice (*Mut* ^−/−^ *MUT* ^4h^) were physically indistinguishable from their *Mut^+/−^* or *Mut^+/+^* litter mates (**Figure 4**) and had a normal life span.

### Urine Analysis

**Figure 6:**
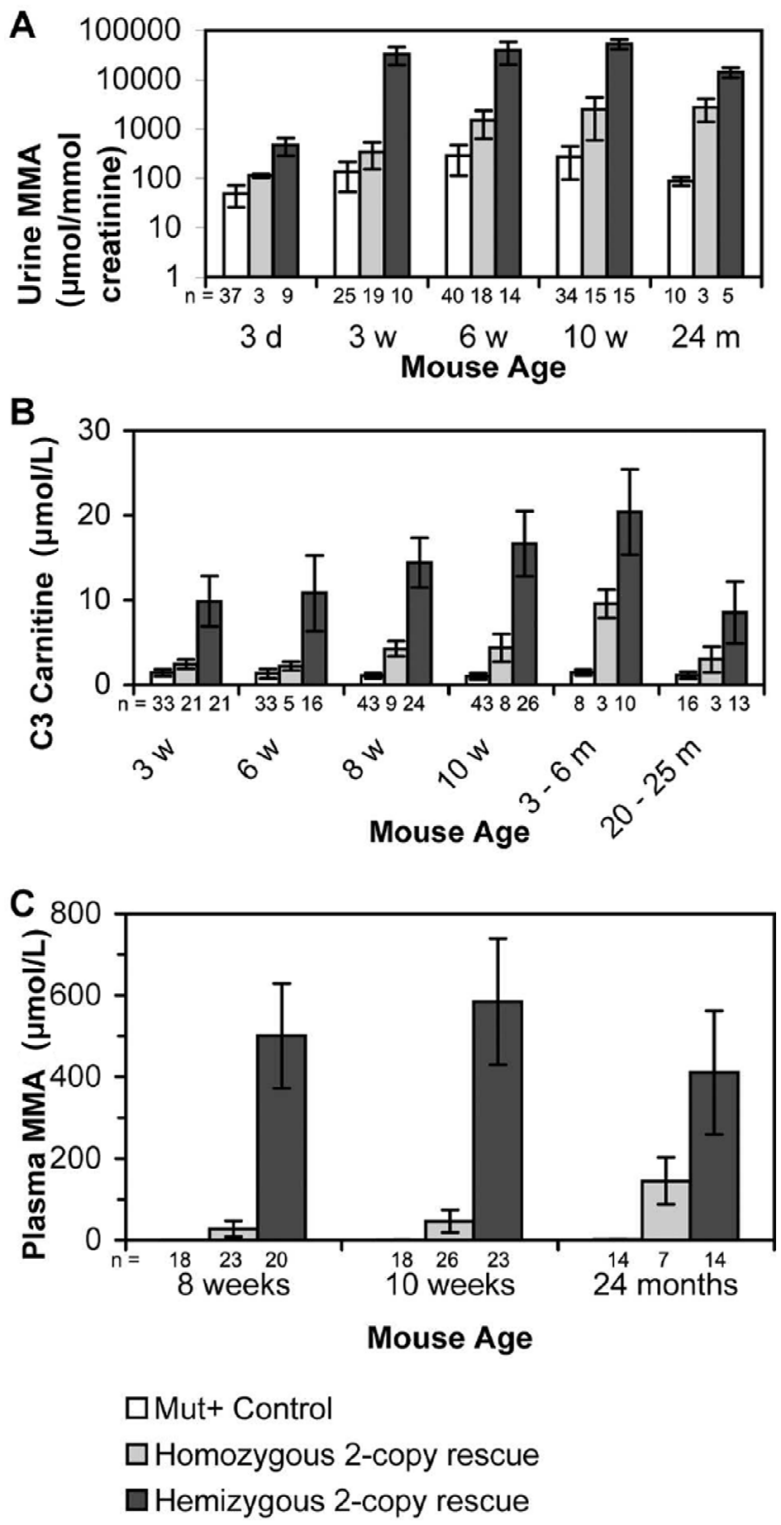
Comparison of metabolite levels from *Mut^+^* controls (*Mut^+/−^* or *Mut^+/+^* ), 2-copy hemizygous rescue and 2-copy homozygous rescue mice at various ages. (A) Methylmalonic acid levels in urine (B) C3 carnitine levels from dried blood spots and (C) methylmalonic acid levels in plasma. Data presented as mean ± SEM. Rescue mice significantly higher (*p*<0.05) in metabolite levels for each sample compared to *Mut^+^* controls.

At three days of age, homo- and hemizygous 2-copy rescue mice demonstrated elevated urine methylmalonic acid levels (two- and ten- fold respectively) in comparison with unaffected *Mut^+/−^* or *Mut^+/+^* littermate controls, which had a urine methylmalonic acid level of 50–290 µmol/mmol creatinine. The urine methylmalonic acid levels increase as the mice aged with a 30- and 160-fold increase at two years of age for homo- and hemizygous 2-copy rescue mice respectively (**Figure 6A**).

The hemizygous 4-copy rescue mice at two years of age had a five-fold increase in urine methylmalonic acid (443±125 µmol/mmol creatinine) compared to control mice which had a mean of 88±17 µmol methylmalonic acid/mmol creatinine. Previous experiments on homozygous knockout pups had demonstrated highest urine methylmalonic acid concentrations of 20,000 µmol/mmol creatinine prior to death [19].

### Acylcarnitine Profile

Analysis of dried blood spots showed a considerable elevation of propionylcarnitine (C3) level in hemizygous and homozygous 2-copy rescue mice for all time points analysed (**Figure 6B**). The 4-copy rescue mice showed a small increase of propionylcarnitine above *Mut^+/−^* or *Mut^+/+^* litter mates (3.0±1.2 compared to 1.2±0.4 µmol/L). The levels of free carnitine (30.3±3.8 µmol/L) and acetylcarnitine (C2) (58.1±12.0 µmol/L) were not significantly changed for any of the mouse models compared to control mice. The ratio of propionylcarnitine to free carnitine (C3:C0) and to acetylcarnitine (C3:C2) of the 2-copy hemizygous partial rescue mice was 9- and 11-fold increased respectively. The homozygous 2-copy rescue mouse had a mean C3 value 3.5-fold higher than the *Mut^+/−^* or *Mut^+/+^* litter mates, with C3:C0 and C3:C2 ratios 3.2- and 3.6-fold higher.

Comparison of control, homozygous knockout and hemizygous 2-copy rescue pup blood prior to birth; at birth; and 16 hours after birth showed that the control pup C3 level does not change significantly after birth (2.0±1.4 µmol/L, n = 38). The homozygous knockout pup C3 level rises from 11.4±3.0 (n = 2) 24 h prior to birth, to 14.1±1.7 (n = 3) at birth, then 16.9±3.7 (n = 10) at 16 hours after birth reaching the maximum of 18.6±6.9 (n* = *5) at death [19]. The hemizygous 2-copy rescue pup blood C3 levels also show an increase, however not as dramatic with a C3 level of 2.3±0.3 µmol/L (n = 2) 24 h prior to birth, 3.0±1.5 (n = 4) at birth, then 3.8±1.5 (n = 4) at 16 hours after birth.

### Plasma and Tissue Methylmalonic Acid Analysis

Analysis of plasma (**Figure 6C**) showed that methylmalonic acid levels were higher in homozygous 2-copy rescue and hemizygous 2-copy rescue mice compared to *Mut^+/−^* or *Mut^+/+^* controls. 3-Hydroxy-butyric acid and lactic acid levels show no significant variation between the samples (data not shown).

**Figure 7:**
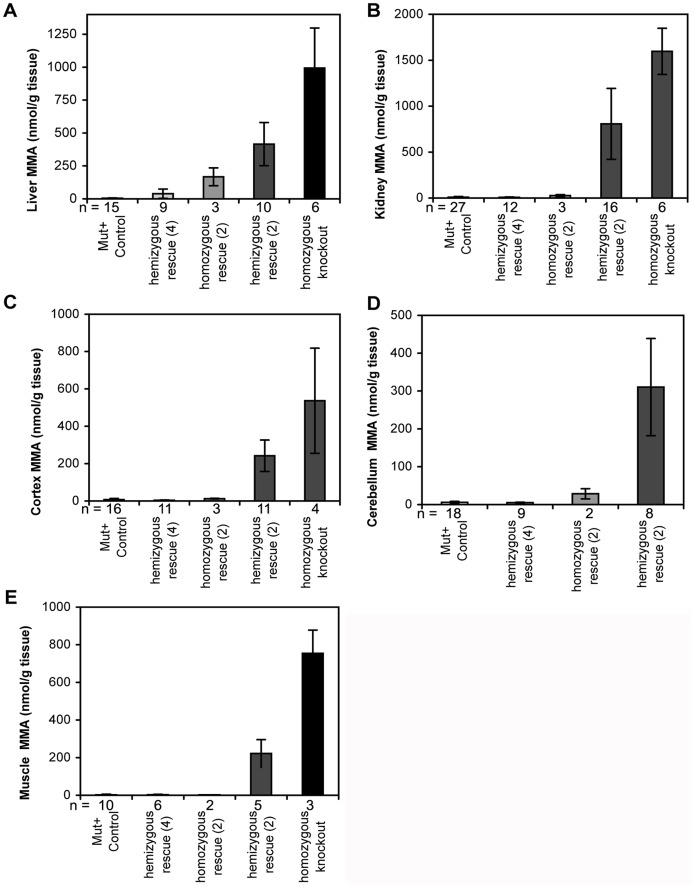
LC-MSMS analysis of methylmalonic acid concentrations in various tissues. (A) liver, (B) kidney, (C) cortex, (D) cerebellum and (E) muscle. Data presented as mean ± SEM.

Tissue methylmalonic acid levels were assessed in adult mouse liver, kidney, cortex, cerebellum and muscle samples (**Figure 7**). The results show the same trend observed in the biochemical analysis of urine and dried blood spots: sharply elevated methylmalonic acid concentrations in hemizygous 2-copy rescue mice and more moderate elevation in the homozygous 2-copy rescue mouse relative to normal litter mates.

### Histological Characterisation

There were no significant abnormalities identified in brain or kidney samples analysed. Detailed histology, performed by the Renal Unit of the Royal Melbourne Hospital, revealed no evidence of interstitial nephritis in either the 4-copy or 2-copy rescue mouse samples analysed (data not shown). Mild fatty changes were observed in the liver of hemizygous 2-copy rescue mice; however these changes were within the normal spectrum.

Detailed histological analysis of liver, kidney and brain in seven week old hemizygous 2-copy rescue mice and two year old hemizygous 2-copy rescue and hemizygous 4-copy mice showed no significant abnormality compared to *Mut^+/−^* or *Mut^+/+^* controls.

### MCM Enzyme Activity

**Figure 8:**
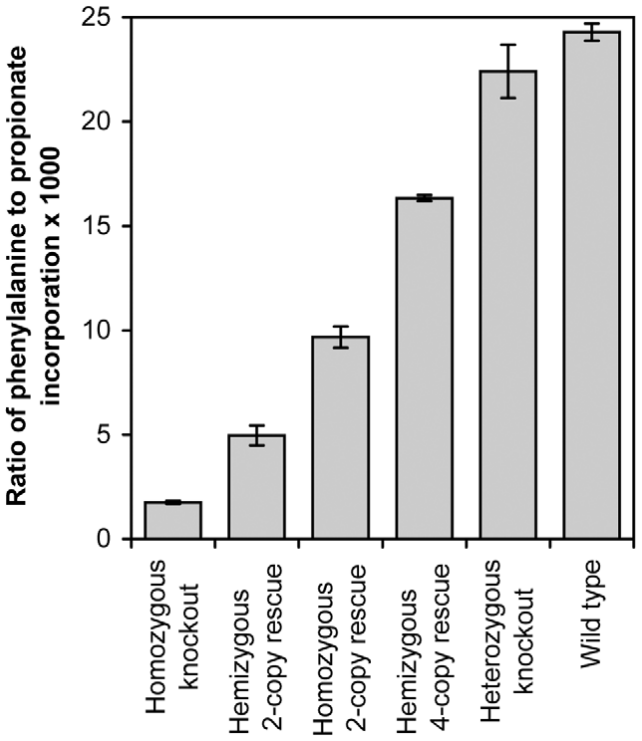
Ratio of incorporation of ^14^C propionate relative to ^3^H phenylalanine incorporation for each mouse cell line. The activity of methylmalonyl-CoA mutase was measured in fibroblast cell lines developed from the mouse models. Homozygous knockout mice with no mouse mutase have a very low phenylalanine to propionate ratio compared to wild type mice. The rescue mouse lines have increasing amounts of enzyme activity compared to the knockout mouse, however do not attain normal levels. Data presented as mean ± SEM.

MCM enzyme activity was assessed indirectly using [^14^C]-propionate incorporation in cultured fibroblasts. The results are presented as a ratio of incorporated propionate to phenylalanine (multiplied by 1000 for ease of analysis) (**Figure 8**). Homozygous knockout mice showed a residual activity of 7% compared to that observed in wild type mice. Hemizygous 2-copy rescue mice had a mean incorporation ratio of 4.96, which is 20% of the wild type control level. The homozygous 2-copy rescue mice showed an incorporation value lower than hemizygous 4-copy rescue mice, despite the same overall copy number with 40% and 67% of wild type incorporation respectively. Incorporation in the heterozygous knockout mouse was 92% that observed in wild type mice.

### RNA Analysis

Liver, kidney and brain samples were collected from wild type, heterozygous transgenic, hemizygous 2-copy rescue, homozygous 2-copy rescue and hemizygous 4-copy rescue mice for RNA extraction. Real time PCR was performed after the RT reaction using the hmMUT and β-actin primers. The hmMUT primers were designed to equally amplify the human and mouse MCM cDNA. Initial testing of these primers concluded that they amplified the mouse and human MCM with 78% and 80% efficiency respectively (data not shown).

**Figure 9:**
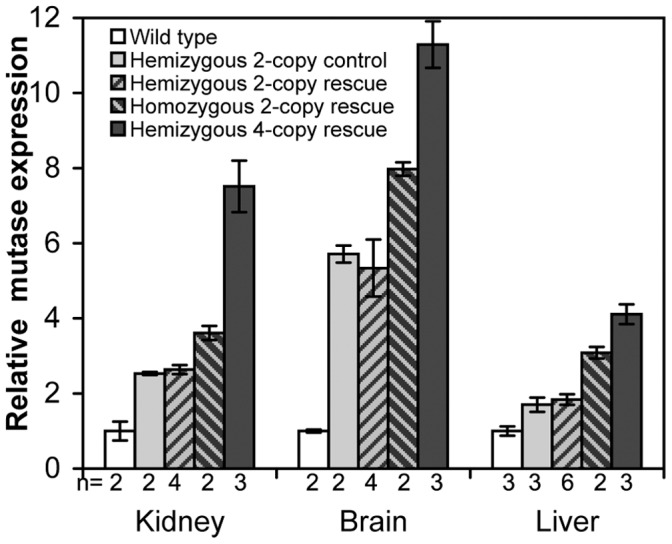
Comparison of mutase expression in the liver, kidney and brain of the 2- and 4-copy mouse models. Expression was normalised to human beta-actin. Data are expressed as mean (± SEM) fold change of mutase expression in each sample relative to wild type mouse mutase expression levels.

MCM mRNA was expressed in all tissues examined (**Figure 9**). The liver showed a 1.8-fold increase from wild type expression in hemizygous 2-copy rescue mice and three- and four-fold increases for homozygous 2-copy rescue and hemizygous 4-copy rescue respectively. The same rise in expression was observed in kidney and brain.

## Discussion

We describe here the production of several transgenic MMA mouse models with the potential to trial therapies for the treatment of MMA. Our aim was to produce transgenic mice with “independent” expression of the human mutase locus i.e. which had the promoter and regulatory sequences for normal expression. It was therefore important to obtain a BAC with the intact human mutase locus and appropriate relatively large flanking regions. After screening of the RPCI-11 BAC library, a BAC of 170 kb was identified that contained 56 kb upstream and 83 kb downstream sequence. The large flanking regions ensure surrounding mutase regulatory elements are present and provide insulating sequence around our gene of interest thereby protecting it from the insertional affects which typically occur with small cDNA inserts. Microinjection of prepared DNA resulted in 6 founder lines, of which four were able to transmit the transgene to the next generation. In the production of transgenic animals, injection of exogenous DNA invariably results in insertion into the chromosome, which in a majority of instances is as a tandem repeat at a single ectopic integration site. Of the six founder mice obtained, it is likely at least three (50%) of the founders were mosaic for the transgene. Support for this was provided by the number of F1 progeny positive for the transgene compared to subsequent progeny (F2 and latter generations) where 50% of progeny were positive. Interphase fluorescent *in situ* hybridisation performed on the 0.6-copy line provided additional support for mosaicism. Furthermore a discrepancy was obtained between the results of transgene copy number from the founders when compared to the progeny in these three lines (**Table 1**).

In humans, the mutase locus behaves as many other mitochondrial genes, with ubiquitous tissue expression and variation in the level of expression between tissues. In keeping with this the human transgene in these transgenic mice has been demonstrated to be expressed in all tissues examined. The amount of human mRNA was examined relative to the endogenous mouse mRNA and has been expressed as a ratio of the two. There is a correlation between copy number dependent expression of the transgene, with lowest levels observed in mice with two copies and highest in mice with six copies of the transgene. Furthermore a tissue dependent pattern of expression was also demonstrated with a similar pattern observed in each of the four lines. The highest expression of the human transgene relative to the endogenous mouse locus, was observed in the brain with lower ratios in the liver and kidney. This would indicate the transgene is functioning in a tissue and copy number dependent manner irrespective of the site of integration supporting the presence of all necessary regulatory elements upon the integrated BAC.

It has been estimated that between 5–15% of transgenic lines cause an insertional mutation [22], [23]. Frequently such chromosomal rearrangements are not recognised until well into breeding when it becomes apparent that either litter sizes are small or mice are being unexpectedly lost. Instances have also been reported where transgene insertions have disrupted an endogenous gene causing an insertional mutation, and compounding observed phenotypes. One founder line (Mouse line E) did not produce any progeny and is presumed sterile, whilst Mouse line C had smaller litter numbers than average. It is possible the integration of the transgene has disrupted a region important for gonadal/gamete production in these mice.

Production of partial rescue mice (mice with no endogenous mouse mutase enzyme activity) showed that two copies of the transgene were sufficient to rescue the neonatal lethal phenotype. The small size and increased loss rate of 2-copy hemizygous rescue mice after weaning would suggest the health of these mice is somewhat compromised; however they are able to survive up to two years. The weight difference of these rescue mice relative to controls is quite striking. The 2-copy hemizygous rescue mice begin life at similar size and weight to their litter mates, however by six weeks there is a significant difference between these two groups. Whilst 2-copy hemizygous rescue mice are smaller in size, some of the difference in weight can be related to the lack of abdominal fat deposits in the rescue mice, which is consistent with the human disorder where anorexia is a problem caused by the metabolic disruption of MMA.

Comparison of mouse organ weight to body weight showed that the kidney, heart, lung and brain of the 2-copy hemizygous rescue mice were significantly larger then expected. Histology of the organs showed no major structural problems. This enlargement may be an artefact caused by the lack of normal body fat distribution, rather than an underlying problem.

Poor growth is a concerning feature in the human disease. Individuals are often below the mean for height, have significant anorexia and may require periods of prolonged nasogastric feeding [7]. As these mice are also well below the mean for weight, they may prove a good model to investigate possible mechanisms for growth impairment, for example growth hormone production. The effect of different dietary regimes may also provide some insight into appropriate treatment.

The crossing of 4-copy mice against heterozygous knockout mice generated mice homozygous for the knockout mutation and hemizygous for the human transgene. These mice, which are referred to as 4-copy rescue mice, ‘rescued’ the neonatal lethality observed in homozygous knockout mice. Interestingly mice homozygous for four copies of the human transgene were not identified despite breeding to produce such offspring. This most likely indicates that the transgene has integrated at a site that may disrupt a critical region which is only observed when homozygosity occurs.

Organic acids are typically cleared from the bloodstream by the kidney such that levels in the urine are significantly higher than blood. The results obtained from the biochemical analysis of the rescue mice reflect this phenomenon. Tandem mass spectrometry was employed to quantitate acylcarnitine levels and methylmalonic acid concentration from blood spots collected onto Guthrie cards. Elevated propionylcarnitine (C3) levels and ratios of C3:C2 and C3:C0 are associated with both propionic aciduria and methylmalonic aciduria. Measurement in the hemizygous 2-copy rescue confirmed the characteristic pattern that is observed in humans affected with MMA: elevation of C3 and elevation of C3:C2 and C3:C0 ratios. Interestingly, homozygous 2-copy rescue were intermediate between hemizygous 2-copy rescue and control mice. The acylcarnitine values obtained in the hemizygous 4-copy rescue mice were normal.

Significant differences exist between the patterns of mutase expression observed in the rescue mice compared to the wild type. The transgenic animals generally showed a similar pattern of expression in kidney, brain and liver. The highest expression of MCM was found in the brain, followed by the kidney then liver. This pattern is contrary to that observed in the wild type with highest levels in the liver, then kidney and brain. These results were reproducible across different samples, extractions and real-time PCR analyses. Furthermore the level of human transgene expression in rescue mice is higher relative to wild type levels than that measured previously in the original transgenic mice with the endogenous mouse locus still functional. It has been suggested that rats do not have the same catabolic pathway for branched chain amino acids as found in humans. In rats, the muscle is the major site of expression of branched-chain amino acid aminotransferase which is the first common step of branched chain amino acid catabolism, whereas the branched chain keto acid dehydrogenase that catalyses the flux-generating step is only very weakly expressed in muscle and mainly expressed in the liver [24]. In contrast, in humans both activities are present in muscle, liver and also in the brain [25]. The whole human *MUT* locus including sequences that putatively regulate the tissue specific expression has been integrated, it is therefore possible that the pattern of the human mutase expression observed in the transgenic mice may reflect the human expression pattern.

Enzymatic measurement of MCM activity was assessed using [^14^C]propionate:[H^3^]phenylalanine incorporation. The higher levels of human transgene expression observed with rescue mice relative to levels obtained in the wild type mice do not translate into higher levels of enzyme activity. This would indicate that either mRNA is inefficiently translated into protein or alternatively the protein is less active or degraded more rapidly. This may reflect interspecies differences in protein biosynthesis or turnover or most likely that the mitochondrial targeting sequence for the human gene is not as efficient at transporting into the mitochondria in the mouse setting. Values obtained in hemizygous 2-copy rescue mice were approximately 20% of wild type values, indicating that just 20% of MCM activity was enough to survive the neonatal lethality observed in the knockout model. Predictably, the gap observed throughout all forms of characterisation between the homozygous 2-copy rescue mouse and the hemizygous 4-copy rescue mouse is present in this analysis also. The homozygous 2-copy rescue mouse has 39.8% activity as opposed to the 67.3% observed in the 4-copy rescue mouse.

The homozygous 2-copy rescue mouse has consistently shown to have a more moderate disease expression than the hemizygous 2-copy mice investigated. Interesting the biochemical parameters investigated and the mRNA and enzyme activity analyses all show a more progressive disease in this mouse compared to 4-copy rescue mice despite the same overall copy number. Whilst use of a “large genomic fragment” should overcome problems associated with position effects, this may not be complete and a degree of gene silencing may be occurring. Alternatively not all copies of the insert may be totally functional.

In summary, these transgenic mice contain the human MMA locus which appears to be fully functional despite a non-targeted site of integration, indicating that the 170 kb fragment contains all the regulatory elements. The sequencing of the human and mouse genomes and rapid technological advances have all contributed the use of mice as an ideal model for human disease. The generation of an MCM knockout mouse [19] was the first step to developing mouse models that enable investigation into the pathophysiology of methylmalonic aciduria and evaluation of novel cell and gene therapy approaches to treatment. Although the knockout mouse is able to reproduce the disease phenotype and biochemical characteristics of the human disease it fails to recapitulate key features at a genotypic and phenotypic level. The new mouse models described here provide an ideal “intermediate” phenotype of MMA disease allowing examination of longer term exposure to high methylmalonic acid levels. Furthermore, as these mice are able to survive the newborn period, they will enable easier study of the effect of liver cell transplantation, viral gene therapy and pharmacological upregulation of gene expression for this disease.

## Materials and Methods

All procedures involving treatment of mice were performed with ethics approval from the Murdoch Childrens Research Institute Animal Ethics Committee (A550). All surgery was performed under isoflurane or avertin anesthesia, and all efforts were made to minimise suffering.

### Transgenic Mouse Production

A fully sequenced 172 kb BAC clone (pBAC_MMA) containing the entire mutase locus and extensive upstream and downstream sequence was identified (RPCI-11-463L20) (**Figure 1**). The BAC was purified and linearised by restriction digestion prior to microinjection into the pronucleus of fertilised C57BL/6 mouse oocytes. Six independent positive transgenic founder mice were identified (two female and four male). Four founders transmitted the intact transgene to offspring.

### Transgene Analysis

To determine transgene integrity, PCR primers were designed one kb in from each end of the BAC RPCI-11-463L20 (BacendF 5′-ACAGTTGGCCTGCTGTATCC-3′, BacendR 5′-CACCATGCCTGGCTAATTTT-3′ and BacstartF 5′-GGCAGAACTTTATGCCAGGA-3′, BacstartR 5′-TGGAGGACCTGAGCTTTCTG-3′).

The transgene insertion site was analysed by fluorescent *in situ* hybridisation using the entire BAC- RPCI-11-463L20 sequence as a probe [26]. The probe was prepared from BAC DNA and labelled with digoxigenin by nick translation according to the manufacturer’s recommendations (Vysis, Abbott Park, IL, USA). Individual metaphase spreads were prepared using fibroblast cultures set up from tail biopsies of the founder mice. The slides were mounted in Vectashield (Vector Laboratories, Burlingame, CA, USA) containing DAPI counter stain. The cells were examined and analysed using a Zeiss epifluorescence microscope (Oberkochen, Germany) with appropriate filters. Images were captured using Cytovision imaging equipment and software (Applied Imaging Corp., Santa Clara, CA, USA).

Transgene copy number was determined for each transgenic mouse founder line. Restriction digestion of genomic DNA from each founder with DraI gave fragments containing exon 2 of the human (1,734 bp) and mouse (3,535 bp) mutase gene. Southern blot probing using an exon 2 PCR product with 100% sequence homology between the human and mouse sequences enabled quantitation of the human band relative to the mouse.

### Comparative Gene Expression Analysis

Total RNA was extracted from various tissues. A competitive RT-PCR technique was performed (forward primer 5′-GGACCATATCCTACCATGTATAC-3′; reverse primer 5′-ACAGTGTAATAGCAACTCCAG-3′) followed by restriction digestion and electrophoresis enabling determination of the amount of RNA expressed from the human transgene relative to the endogenous mouse locus. The primers equally amplified the human and mouse transcripts and gave a product of 222 bp. The human specific product was distinguished by restriction digestion with XhoI, to produce 174 bp and 84 bp fragments.

### Production of Transgenic Mice on a Knockout Background

The 2-copy (*MUT* ^2h^) (0.6-copy founder line) and 4-copy (*MUT* ^4h^) (2-copy Anne founder line) hemizygous transgenic mice were mated with heterozygote mouse MCM knockout (*Mut* ^+/−^) mice. These F1 *Mut* ^+/−^ *MUT* ^2h^ and *Mut* ^+/−^ *MUT* ^4h^ mice were then crossed with heterozygous knockout mice to produce rescue mice: human transgene present on a knockout background (*Mut* ^−/−^ *MUT* ^2h/2h^ and *Mut* ^−/−^ *MUT* ^4h/4h^).

### Multiplex PCR Analysis of Offspring

Screening of the mouse pups was performed using multiplex PCR on tail genomic DNA. The following primer sets were used: Mouse *Mut* forward (5′-CTATTCTGTTGCTTTTATT ATTGT-3′) and Mouse *Mut* reverse (5′-GAAAAATATAAGTATTTCTGACCAT-3′), Neo/Kan forward (5'-ATGATTGAACAAGATGGATT-3') and Neo/Kan reverse (5'-GCCATGATGGAT ACTTTCT-3'), Human *MUT* forward (5′-CAGGGTTTTTATA GTCATTA-3′) and Human *MUT* reverse (5′-CAAGATTCCCATCACAGT-3′). The primers produce products of 507, 350 and 276 bp respectively.

### Metabolite Analysis and Enzyme Activity Assay

Urine methylmalonic acid levels were measured by LC-MSMS as previously described [19]. Creatinine levels were measured for normalisation of urine diluteness. This was performed by the diagnostic laboratory of the VCGS (Murdoch Childrens Research Institute, Parkville, VIC, Australia) using a standard Jaffe method.

Blood was spotted onto absorbent cotton fibre paper (Guthrie) card at various ages for analysis of acylcarnitine levels. After drying, 0.125 inch spots were excised and their contents extracted with methanol and butylated in microtitre plates and analysed by direct injection MSMS according to standard methods.

The plasma methylmalonic acid analysis method was adapted from a method established by Magera *et al.* [27] and includes the following modifications. Fifty microlitres of plasma was pipetted into wells of a 96-well plate. Methanol (200 µL) containing 0.5 µmol/L internal standard (^2^H_3_-methylmalonic acid; Cambridge Isotope Laboratories, Andover, MA) was added to each sample. The protein precipitate was spun down by centrifuging at 350 *g* for three minutes. The supernatant was then removed and dried under an air stream at 65°C. The samples were butylated with 50 µL of n-butanol:acetyl chloride (9∶1). The plate was shaken for 20 min, heated at 65°C for 20 min and then neutralised in 200 µL of neutralising buffer (100% methanol, 0.5 mol/L ammonium acetate pH 9.0).

Samples were run using a 5 µm, Hypersil ODS 2.1×100 mm C18 column (Hewlett Packard). The mobile phase consisted of 20% Buffer A (9∶1 water: 0.2 ammonium formate, pH 3.5) and 80% Buffer B (9∶1 methanol: 0.2 ammonium formate, pH 3.5) run with a flow rate of 0.2 mL/min. The transitions *m/z* 231 to *m/z* 119 and *m/z* 234 to *m/z* 122 were used in the selected reaction monitoring mode for methylmalonic acid and ^2^H_3_-methylmalonic acid respectively.

Tissue methylmalonic acid analysis was performed on frozen tissue samples that were weighed precisely and homogenised in 500 µL of water. Samples were then sonicated and spun at 800 *g* for 10 min. Supernatant (50 µL) was then pipetted into a 96-well plate and prepared for analysis as for plasma samples. Samples were then analysed under the same LC-MSMS conditions as used for plasma methylmalonic acid analysis.

MCM activity within mouse fibroblast cell lines was determined by measuring the incorporation of [^14^C]propionate into trichloroacetic acid-precipitable material [28] and expressed as a ratio to [H^3^]phenylalanine incorporation [20].

### Real-time RT-PCR Analysis

Reverse transcribed cDNA corresponding to 25 ng of RNA was amplified in 25 µL total volume of PCR reaction mix containing ABsolute™ QPCR SYBR® Green mix (ABgene, Epsom, Surrey, UK) PCR MasterMix, 0.5 µL ROX and 80 nM forward and reverse primers. Primers were designed to amplify the human and mouse MCM gene with equal efficiency. Sequences were as follows: forward 5′-TTCTATAAGGACAAC ATTAAGGCTGGTC-3′, reverse 5′-CAATAGCAACTCCAGCCATTCC-3′. Primers which amplify a section of the mouse housekeeping gene, *ACTB* (β-actin) were used as an internal control (forward 5′-CCCTAAGGCCAACCGTGAA-3′ and reverse 5′-CAGCCTGGATGGCTACGTACA-3′). Cycle threshold values of *Mut/MUT* mRNA were normalised to the amount of *ACTB* mRNA measured by real-time PCR in each sample (assayed in triplicate). The results were analysed using the “delta-delta comparative Ct method” and are given as relative mutase level compared to wild type mice.

### Statistical Analysis

Data are expressed as means ± SEM. Statistical analysis was performed by one-way analysis of variance (ANOVA). Statistical significance was accepted at *p*<0.05.

## References

[1] Chace DH, DiPerna JC, Kalas TA, Johnson RW, Naylor EW (2001). Rapid diagnosis of methylmalonic and propionic acidemias: quantitative tandem mass spectrometric analysis of propionylcarnitine in filter-paper blood specimens obtained from newborns.. Clin Chem.

[2] Willard HF, Rosenberg LE (1980). Inherited methylmalonyl CoA mutase apoenzyme deficiency in human fibroblasts: evidence for allelic heterogeneity, genetic compounds, and codominant expression.. J Clin Invest.

[3] Lindblad B, Lindblad BS, Olin P, Svanberg B, Zetterstrom R (1968). Methylmalonic acidemia. A disorder associated with acidosis, hyperglycinemia, and hyperlactatemia.. Acta Paediatr Scand.

[4] Matsui SM, Mahoney MJ, Rosenberg LE (1983). The natural history of the inherited methylmalonic acidemias.. N Engl J Med.

[5] Feillet F, Bodamer OA, Dixon MA, Sequeira S, Leonard JV (2000). Resting energy expenditure in disorders of propionate metabolism.. J Pediatr.

[6] Hyman SL, Porter CA, Page TJ, Iwata BA, Kissel R (1987). Behavior management of feeding disturbances in urea cycle and organic acid disorders.. J Pediatr.

[7] Kahler SG, Millington DS, Cederbaum SD, Vargas J, Bond LD (1989). Parenteral nutrition in propionic and methylmalonic acidemia.. J Pediatr.

[8] Leonard JV (1995). The management and outcome of propionic and methylmalonic acidaemia.. J Inherit Metab Dis.

[9] Leonard JV, Vijayaraghavan S, Walter JH (2003). The impact of screening for propionic and methylmalonic acidaemia.. Eur J Pediatr.

[10] Yannicelli S, Acosta PB, Velazquez A, Bock HG, Marriage B (2003). Improved growth and nutrition status in children with methylmalonic or propionic acidemia fed an elemental medical food.. Mol Genet Metab.

[11] Al Essa M, Rahbeeni Z, Jumaah S, Joshi S, Al Jishi E (1998). Infectious complications of propionic acidemia in Saudia Arabia.. Clin Genet.

[12] Church JA, Koch R, Shaw KN, Nye CA, Donnell GN (1984). Immune functions in methylmalonicaciduria.. J Inherit Metab Dis.

[13] Corazza F, Blum D, Clercx A, Mardens Y, Fondu P (1996). Erythroblastopenia associated with methylmalonic aciduria. Case report and in vitro studies.. Biol Neonate.

[14] Inoue S, Krieger I, Sarnaik A, Ravindranath Y, Fracassa M (1981). Inhibition of bone marrow stem cell growth in vitro by methylmalonic acid: a mechanism for pancytopenia in a patient with methylmalonic acidemia.. Pediatr Res.

[15] Kahler SG, Sherwood WG, Woolf D, Lawless ST, Zaritsky A (1994). Pancreatitis in patients with organic acidemias.. J Pediatr.

[16] Threadgill DW, Wilkmeyer M, Womack JE, Ledley FD (1990). Localization of the murine methylmalonyl CoA mutase (Mut) locus on chromosome 17 by in situ hybridization.. Cytogenet Cell Genet.

[17] Wilkemeyer MF, Crane AM, Ledley FD (1990). Primary structure and activity of mouse methylmalonyl-CoA mutase.. Biochem J.

[18] Wilkemeyer MF, Andrews ER, Ledley FD (1993). Genomic structure of murine methylmalonyl-CoA mutase: evidence for genetic and epigenetic mechanisms determining enzyme activity.. Biochem J 296 (Pt.

[19] Peters H, Nefedov M, Sarsero J, Pitt J, Fowler KJ (2003). A knock-out mouse model for methylmalonic aciduria resulting in neonatal lethality.. J Biol Chem.

[20] Buck NE, Wood L, Hu R, Peters HL (2009). Stop codon read-through of a methylmalonic aciduria mutation.. Mol Genet Metab.

[21] Hu R, Buck NE, Khaniani MS, Wood L, Wardan H (2009). Gene induction for the treatment of methylmalonic aciduria.. J Gene Med.

[22] Nagy A, Gertsensten M, Vintersten K, Behringer R (2003). Manipulating the mouse embryo: a laboratory manual..

[23] Le Saux A, Houdebine L-M, Jolivet G (2010). Chromosome integration of BAC (bacterial artificial chromosome): evidence of multiple rearrangements.. Transgenic Res.

[24] Suryawan A, Hawes JW, Harris RA, Shimomura Y, Jenkins AE (1998). A molecular model of human branched-chain amino acid metabolism.. Am J Clin Nutr.

[25] Brosnan JT, Brosnan ME (2006). Branched-chain amino acids: enzyme and substrate regulation.. J Nutr.

[26] Howden SE, Voullaire L, Wardan H, Williamson R, Vadolas J (2008). Site-specific, Rep-mediated integration of the intact beta-globin locus in the human erythroleukaemic cell line K562.. Gene Ther.

[27] Magera MJ, Helgeson JK, Matern D, Rinaldo P (2000). Methylmalonic acid measured in plasma and urine by stable-isotope dilution and electrospray tandem mass spectrometry.. Clin Chem.

[28] Willard HF, Ambani LM, Hart AC, Mahoney MJ, Rosenberg LE (1976). Rapid prenatal and postnatal detection of inborn errors of propionate, methylmalonate, and cobalamin metabolism: a sensitive assay using cultured cells.. Hum Genet.

